# Serum and Urine Metabolites in Healthy Men after Consumption of Acidified Milk and Yogurt

**DOI:** 10.3390/nu14224794

**Published:** 2022-11-12

**Authors:** Ueli Bütikofer, René Badertscher, Carola Blaser-Freiburghaus, Pascal Fuchsmann, Mireille Tena Stern, Philipp A. Kuert, Grégory Pimentel, Kathryn Jane Burton-Pimentel, Nathalie Vionnet, Guy Vergères

**Affiliations:** 1 Agroscope, Schwarzenburgstrasse 161, 3003 Bern, Switzerland; 2 Service of Endocrinology, Diabetes and Metabolism, Lausanne University Hospital, 1005 Lausanne, Switzerland

**Keywords:** metabolomics, dairy, yogurt, milk, postprandial, fermentation, healthy men, nutrivolatilomics

## Abstract

The identification of molecular biomarkers that can be used to quantitatively link dietary intake to phenotypic traits in humans is a key theme in modern nutritional research. Although dairy products (with and without fermentation) represent a major food group, the identification of markers of their intake lags behind that of other food groups. Here, we report the results from an analysis of the metabolites in postprandial serum and urine samples from a randomized crossover study with 14 healthy men who ingested acidified milk, yogurt, and a non-dairy meal. Our study confirms the potential of lactose and its metabolites as markers of lactose-containing dairy foods and the dependence of their combined profiles on the fermentation status of the consumed products. Furthermore, indole-3-lactic acid and 3-phenyllactic acid are two products of fermentation whose postprandial behaviour strongly discriminates yogurt from milk intake. Our study also provides evidence of the ability of milk fermentation to increase the acute delivery of free amino acids to humans. Notably, 3,5-dimethyloctan-2-one also proves to be a specific marker for milk and yogurt consumption, as well as for cheese consumption (previously published data). These molecules deserve future characterisation in human interventional and observational studies.

## 1. Introduction

Fermented foods such as cheese, yogurt, bread, or sauerkraut have been consumed worldwide and in great variety for thousands of years throughout human history. On the technological side, methods to prepare these foods evolved from spontaneous fermentation to the selection of starter cultures with known properties [1]. Lactic acid bacteria are among the most important microorganisms in the fermentation of foods [2,3]. These bacteria are able to convert carbohydrates such as lactose into lactate and are now widely used for commercial food production (yogurt, cheese, sausages, olives, chocolate, etc.).

In addition to extending the shelf life of foods and providing the desired development of texture and flavour, fermentation can change the composition of the food with increasing studies exploring novel potential health benefits associated with these changes [4]. For example, from a nutritional perspective, proteolysis in fermented dairy products leads to an increased release of bioactive peptides [4]. In addition, people with lactose intolerance can consume fermented dairy products, as a large proportion of the lactose in these products has been hydrolysed to glucose and galactose by bacterial β-galactosidases [5]. The positive effects of fermented foods on human health extend beyond the source materials of the food [6,7,8] and may include hypocholesterolemic, antioxidant, bone-strengthening, blood pressure-lowering, anticarcinogenic, immunomodulatory, and antiallergenic effects modulated by their impact on the composition and dynamics of the gut microbiota. Many of these effects can be attributed to bioactive substances that are directly produced from the food during the technological fermentation process or indirectly produced in the gastrointestinal tract following the transformation of nutrients by the intestinal microbiota [9,10,11].

A promising approach to deciphering the complex interactions between fermented foods and humans is untargeted metabolomics, using high-resolution chromatographic methods [12,13]. Metabolomics is a powerful analytical strategy that allows the identification of biomarkers of food intake (BFI), of the effect of food on the human organism, and of the susceptibility—that is, the inter-individual variability—of the metabolic response of humans to food intake [14]. However, comprehensive studies that determine the complete or nearly complete set of metabolites derived from the food fermentation process and present in body fluids as a result of their metabolic processing by humans are still rare.

Given the wealth of research available on the milk matrix, dairy products, such as cheese [15,16] or yogurt [17,18] are interesting model products for investigating the impact of food fermentation on the metabolic response of the organism. Several candidate BFIs have previously been reported in human intervention studies with milk, cheese, and yogurt, using a range of metabolomics platforms, including LC-MS, GC-MS, and NMR, with both blood and urine samples [15,16,17,18]. However, compared to other food groups, dairy products with and without fermentation remain poorly characterised with regard to markers of intake [19]. In the present study, metabolites of postprandial serum and urine were measured by GC-MS after the consumption of acidified milk and probiotic yogurt by healthy adult men in a double-blinded crossover clinical intervention. The fermented milk product was a yogurt containing the probiotic *Lactobacillus rhamnosus* Gorbach-Goldini (LGG) with immunomodulatory properties, and acidified milk was used as the non-fermented control. A non-dairy high-fat meal (HFM) was used to compare postprandial metabolites to dairy products. Two sample preparation and extraction methods were used: a classical chemical derivatisation method with GC-MS separation, which yields complementary information to previous LCMS results [17], and a recently developed headspace method, which directly extracts volatile compounds (volatilomics) and expands the number of molecules identified by GC-MS-based metabolomics beyond the classical method with derivatisation [20]. This combination of methods consequently allowed the confirmation of candidate markers of fermented dairy products as well as the discovery of new, so far unreported, BFIs.

## 2. Materials and Methods

### 2.1. Subjects

The subjects were healthy adult men (*n* = 14) aged 24.6 ± 4.7 years (mean ± SD) with a BMI of 21.8 ± 1.8 kg/m^2^. They had no intolerance or adverse reactions to dairy products. For further details on exclusion criteria and inclusion visits, see Burton et al. [21]. One subject was excluded from all analyses because of suspected noncompliance with dietary instructions. Another subject withdrew before the last day of testing because of an acute illness. Before the start of the study, all subjects gave written informed consent.

### 2.2. Test Products

Probiotic yogurt (referred to as yogurt hereafter) was prepared by fermentation of milk with classical yogurt starter cultures and the widely used probiotic LGG. The starter cultures consisted of a mixture of *Lactobacillus delbrueckii* ssp. *bulgaricus* and *Streptococcus thermophilus* and were purchased from Chr. Hansen A/S, Denmark (Thermophilic Yoflex, Hoersholm, Denmark). Probiotic LGG was obtained from the Culture Collection of the University of Gothenburg, Sweden (CCUG 34.291). All milk used in this study was obtained from Emmi (Mittelland Molkerei AG, Suhr, Switzerland) from a single production batch of full-fat homogenised, ultra-high-temperature-treated milk. Chemically acidified milk (referred to as milk hereafter) was prepared by adding 2% 1,5-gluconolactone (GDL, ≥99.0%, Jungbunzlauer AG, Basel, Switzerland) to the full-fat milk. The addition of 2% GDL to milk mimics the slow pH reduction that occurs during the classical fermentation of yogurt and allows the production of a mild semi-liquid yogurt imitate. Details of product preparation, nutrient composition, and biochemical analyses are given in a publication by Pimentel et al. [17]. The HFM consisted of bread, palm fat, salami, and boiled eggs (53% energy from fat), as described previously. The HFM challenge test was originally used to compare the postprandial inflammatory response after the 2-w intake of the probiotic yogurt compared with the acidified milk [21].

### 2.3. Study Design

The study used a randomised double-blind crossover design to compare the two dairy products. The postprandial response to 800 g of the two test dairy products, milk (2352 kJ) and yogurt (2120 kJ), was assessed at the beginning of each intervention phase. The subjects ingested 800 g of the test product within 15 min. Blood serum was sampled postprandially at 0 (fasting, pre-challenge), 15, 30, 60, 90, 120, 180, 240, 300, and 360 min [21]. After ingestion of 292 g HFM (4120 kJ), blood serum was sampled postprandially at 0, 60, 120, 180, 240, 300, and 360 min. To reduce the resources needed to measure the samples, the number of analyzed samples was reduced after consumption of the HFM. Serum samples were kept at −80 °C prior to analysis. Baseline ‘spot’ urine was collected upon arrival at the study centre and pool urine samples were collected over the 6 h test period. A detailed graphical overview of the study design was already published [21]. The volume of each urine collection was recorded; the samples were transferred into smaller tubes, centrifuged at 1800× *g* for 10 min at 4 °C, and stored at −80 °C. During the two weeks following the dairy challenge test, the participants consumed 400 g of the test product each day to estimate the impact of each test product on fasting serum metabolites measured by LCMS [17]. Dietary intake was semi-controlled during all phases of the study. Only dairy products provided by the study organisers were allowed to be consumed. There were special instructions for the consumption of fermented foods, alcohol and caffeine to maintain the participants’ individual dietary patterns. Prior to each test, the subjects were instructed to follow a three-day controlled diet with non-dairy and non-fermented foods provided by the investigators, as described previously [21]. 

### 2.4. GC-MS Analysis with Derivatisation

#### 2.4.1. Sample Preparation

For food analysis, 2.4 g of food was mixed with 20 mL of ultrapure water (Milli-Q^®^ IQ 7000, Merck, Darmstadt, Germany), and the supernatant was collected after centrifugation at 1800× *g* for 10 min at 4 °C. All test product samples (100 μL) were precipitated with cold methanol (300 μL) prior to derivatisation, and the supernatant was transferred to a new tube and dried using a vacuum centrifuge.

The preparation of the serum samples was based on Dunn et al.’s [22] method, with few modifications. A volume of 50 μL internal standard solution (0.20 g/L) (labelled D-fructose, U-13C6, 99%, Cambridge Isotope Laboratories, Inc., UK) was added to 100 μL serum samples and then precipitated with 300 μL of cold methanol. The suspension was centrifuged at 1800× *g* for 10 min at 4 °C, and the supernatant (370 μL) was transferred to a new tube and dried using a vacuum centrifuge.

The 6 hour pooled urine samples were normalised by diluting with Milli-Q^®^ water to reach the same specific gravity (1.0008). This normalisation procedure reduces the loss of information due to the large differences in the initial concentration [23]. To 100 μL of urine sample, 50 μL of internal standard solution (0.17 mg/mL) labelled D-fructose (U-13C6, 99%, Cambridge Isotope Laboratories, Inc., Cambridge, UK) were added and dried using a vacuum centrifuge.

#### 2.4.2. Sample Analysis

The dried serum, food and urine extracts were two-step derivatised (methoximation and trimethylsilylation) and injected into a multimode injector using the following temperature program: initially 90 °C, heating rate 900 °C/min until 280 °C, cooled for 5 min at a rate of 30 °C/min, and kept at 250 °C. The separation was performed on a GC-MS system (Agilent 7890B/MS5977A, Agilent Technologies, Santa Clara, CA, USA) equipped with a DB-5 ms fused silica capillary column (60 m, 0.25 mm i.d., 0.25 μm film thickness, Agilent Technologies, Basel, Switzerland) and coupled to a CombiPAL autosampler (CTC-Analytics AG, Zwingen, Switzerland). The temperature programming of the GC oven was: initial temperature 70 °C for 2 min, increase up to 160 °C at a rate of 5 °C/min, increase to 300 °C at a rate of 10 °C/min, which was held for 16 min, equilibration time 1 min. The MS detection mass ranged from 28.5 to 600 Da, the MS source temperature was 230 °C, and the MS Quad temperature was 150 °C. Electron ionisation was performed at 70 eV.

A batch consisted of all samples from one subject, the order of injection of each batch was randomized. Serum and urine samples were randomised separately for measurement using the Excel function RAND. Quality control samples (QC) were prepared beforehand by mixing all serum or urine samples separately at equal volumes. Each batch was initiated by five injections of QC samples for equilibration, and after every fifth serum sample, fresh QC was injected. At the start and end of one batch, a blank sample (pure water) was included. QC samples and blank samples underwent the same preparation as the serum samples.

#### 2.4.3. Data Pre-Treatment

Raw data files were deconvoluted and converted into CFE files using Masshunter Profinder Software (Agilent Technologies, Santa Clara, CA, USA) in recursive mode. The first identification of GC-MS metabolomics data was done using the GOLM Metabolome database [24]. GC-MS data were filtered by removal of features with low frequency detected in <50% of all QCs. All compounds with the functional group names dioxane, dioxolane, dioxol, silane, and silanol as their main designations were removed, as they were likely contaminants originating from the GC column. Further, alkanes (decane, dodecane, pentadecane, nonadecane docosane) and octanoic acid were removed, as they likely originated from the consumable used for sample preparation and storage.

Postprandial active features were analysed using MassHunter Quantitative Analysis (Agilent Technologies, Santa Clara, CA, USA) in the targeted mode. Peak integration was checked for each metabolite individually. The results of the quantifier ion were normalised with the internal standard D-fructose (U-13C6, 99%, Cambridge Isotope Laboratories, Inc., Cambridge, UK).

### 2.5. Nutrivolatilomics Analysis

#### 2.5.1. Sample Preparation

Urine and serum samples were concentrated by solid phase extraction (SPE). Prior to use, the SPE cartridges were conditioned with 3 × 1 mL hexane, 3 × 1 mL acetonitrile, 3 × 1 mL Milli-Q^®^ water, and 1 × 1 mL phosphoric acid 4%.

The serum samples were shaken on a vortex, and 250 μL were collected and acidified with 500 µL of phosphoric acid 4% in Milli-Q^®^ water. The samples were mixed using the vortex and concentrated through a SPE cartridge Chromabond HR-X, 45 µm (Macherey-nagel, Oensingen, Switzerland), 1 mL/30 µg. After the drying stage (15 min under nitrogen flow), elution was performed with 300 µL of acetonitrile directly into 20 mL headspace vials. The vials were hermetically sealed with a silicone-Teflon septum (Macherey-Nagel, Oensingen, Switzerland) and stored at 4 °C until analysis.

Urine samples were thawed overnight at 4 °C in the refrigerator. Pooled 6 h urine samples prepared for GC-volatile analyses were normalised based on the lowest specific gravity of all samples (1.0008), as described previously [16] (see also above). Urine samples were pre-concentrated using a SPE cartridge. The protocol for sample preparation has been presented elsewhere [25] and was applied to the samples, with minor modifications. From the pool samples, 500 μL of urine was acidified to pH 2.0 by adding 500 μL phosphoric acid 4% and then concentrated on an SPE cartridge Chromabond HR-X, 45 µm, 1 mL/30 µg. The cartridges were dried, as with the serum protocol, and eluted with 300 μL acetonitrile in 20 mL headspace vials. The vials were hermetically sealed with a silicone-Teflon septum and stored at 4 °C until analysis.

#### 2.5.2. Sample Analysis

A MPS2 autosampler (Gerstel, Sursee, Switzerland) and an Agilent 7890B gas chromatography (GC) system coupled with an Agilent 5977A mass selective detector (MSD) (Agilent Technology, Santa Clara, CA, USA) were used for volatile analyses. A batch consisted of all samples from one subject, the order of injection of each batch was randomized. Serum and urine samples were randomised separately for measurement using the Excel function RAND. The samples were extracted by Dynamic Headspace Vacuum transfer in Trap extraction (DHS-VTT) according to the method described by Fuchsmann et al. [20]. For urine and serum samples, the headspace of the vial was incubated for 5 min and extracted without agitation for 5 min at 60 °C under vacuum (5 mbar) using a vacuum pump Buchi V-300 (Buchi, Flawil, Switzerland) and in-tube extraction (ITEX) materials equipped with a trap filled with Cabosieve S III/Tenax TA (ITEX2, Brechbühler, Switzerland) as previously described [20]. The bound volatiles were desorbed from the sorbent for 2 min under a nitrogen flow of 220 mL/min at the recommended temperature for the employed polymer (300 °C) in a programmed temperature vaporiser (PTV) injector in vent mode at 50 mL/min and 20 kPa for 120 s. The injector was equipped with a glass liner filled with Tenax TA and cooled with liquid nitrogen at 10 °C. The injector was then heated at a rate of 12 °C/s, to 240 °C. The purge flow to the split vent was set at 100 mL/min after 2 min. The reconditioning of the trap was achieved at 300 °C under a nitrogen flow of 100 mL/min for 15 min. Volatile compounds were separated on an Optima FFAP-Plus fused silica capillary column (100% polyethylene glycol PEG with nitroterephthalic acid, bonded, and cross-linked, 30 m × 0.32 mm × 1.0 μm film; Macherey-Nagel, Oensingen, Switzerland) with helium as the carrier gas at a constant flow of 1.3 mL/min (velocity 20 cm/s). The oven temperature was programmed as follows: 5 min at 40 °C, then heated to 210 °C at a rate of 3 °C/min with a final hold time of 12 min (total run time: 73 min). The MS settings were as follows: transfer line at 230 °C and source temperature at 230 °C. The analytes were monitored in SCAN mode between 29 and 300 amu with a gain of 15 and a solvent delay of less than 3 min. The autosampler was controlled with Cycle Composer V. 1.5.4 (CTC Analytics, Zwingen, Switzerland) and the CIS 4 injector with Maestro1 software V.1.4.8.14/3.5 (Gerstel GmbH & Co. KG, Mülheim an der Ruhr, Germany).

The volatilome of the three food products was not investigated, given that the efficiency of the headspace extraction of the volatile compounds was expected to be influenced by the composition and properties of the food matrices, hence not allowing a relative comparison of the content of the features between them.

#### 2.5.3. Data Pre-Treatment

Masshunter Profinder software version 10.0 in recursive mode and Profiler (Agilent Technologies, Santa Clara, CA, USA) were used for the deconvolution and grouping of the MS signals. Deconvolution was based on five main parameters (retention time tolerance: ±0.3 min, peak height: >1500 ion counts, minimum dot product value: 0.4, integration mode: Agile 2, and smoothing: Gaussian). Features with an ion count < 3 times the median height of the average background noise were excluded during deconvolution. Postprandial active features were analysed using MassHunter Quantitative Analysis (Agilent Technologies, Santa Clara, CA, USA) in the targeted mode. Peak integration was done manually for each metabolite individually.

### 2.6. Statistical Analyses

The data evaluation in this study used non-parametric robust statistical tests, as many variables did not have a normal distribution. All data were processed in the R environment (4.0.1) [26]. The incremental area under the curve (iAUC) for serum results was calculated by cumulative multiplication of the time intervals with mean intensity levels (corrected with internal standards and minus the pre-challenge intensity at t = 0 min) from the deconvoluted results with Masshunter Profinder. The missing values were replaced by the average values of the adjacent values. The iAUC was calculated for each feature in the postprandial phase using the R MESS package (version 0.5.7) [27]. The results of the 6 hour urine pool were directly used for statistical tests.

Postprandial active variables in serum were determined using the Wilcoxon signed-rank test of the serum iAUC of all features. For this purpose, we tested whether the iAUC in serum was significantly different from zero after consumption of milk or yogurt. Control of false positive discovery rate (FDR) was performed with Benjamini–Hochberg correction (*p* < 0.10) [28].

The MS signals of all sample injections of the postprandial active compounds that could be identified at levels 1 (compounds were identified by comparison to a pure reference (injection) and 2 (compounds were identified based on a spectral database) [29] were manually reintegrated (Table 1 and Table 2). After reintegration, a Wilcoxon signed-rank test (*p* < 0.05) was again performed to test whether the iAUC of each feature was significantly different from zero after consumption of milk or yogurt to confirm it as a postprandial active variable. To compare the postprandial effects of milk and yogurt (iAUC and 6 hour urine pool), a paired Wilcoxon signed-rank test was applied to the reintegrated results. Differences with a *p*-value < 0.05 were considered statistically significant.

A third paired Wilcoxon signed-rank test (*p <* 0.05) was applied on the reintegrated compounds to test whether the fasting serum results before milk and yogurt intake were significantly different.

The comparison of the test products milk (*n* = 5) and yogurt (*n* = 5) was performed with the Wilcoxon signed-rank test. Differences with a *p*-value < 0.05 were considered statistically significant.

Lastly, a Wilcoxon signed-rank test (*p <* 0.05) was applied to test whether the iAUC of the HFM was significantly different from 0 to confirm it as a postprandial active variable for this non-dairy challenge.

Hierarchical clustering analysis was conducted on the median values of the final serum dataset to group metabolites based on their postprandial kinetics (amap and dendextend R packages, clustering by Euclidean distance measure and Ward linkage). Five clusters were chosen based on the distance of the clusters.

## 3. Results and Discussion

### 3.1. Overview of the GC-MS Results with Derivatisation

After data deconvolution with Masshunter, a total of 16,058 features in the serum were integrated. However, only 520 features were present in more than 50% of the QCs. After Wilcoxon rank sum test and FDR correction (*p* < 0.1), 487 features were confirmed as ‘postprandial active’ after milk or yogurt intake; 220 features (45%) showed a significant postprandial effect after milk intake, 178 features (37%) showed a significant postprandial effect after yogurt intake, and 89 features (18%) showed a significant postprandial effect after both milk and yogurt intake. Among the significant features, 26 postprandial active compounds could be identified at levels 1 or 2 and were manually reintegrated (selected qualifier and quantifier ions and defined retention indices) for the final statistics (Table 1). Four additional compounds (glutamine, galactonic acid, pyruvic acid, and aspartic acid) were already characterised in previous studies as postprandial active compounds, thus present in the internal database and identified at levels 1 or 2, and were also reintegrated for the final statistics (Table 1). After reintegration, the iAUC of 27 compounds remained significantly different from zero (*p* < 0.05) after milk or yogurt intake.

A comparison of the fasting results in serum before the intake of milk or yogurt did not show any significant difference between the two product groups for any of the 30 manually reintegrated compounds.

The largest group of postprandial active compounds with 21 molecules was composed of amino acids and their derivatives. In addition, we quantified five carbohydrates and their derivatives, two fatty acids, and two molecules resulting from the use of 1,5-gluconolactone, the acidifying agent in milk. 

**Figure 1:**
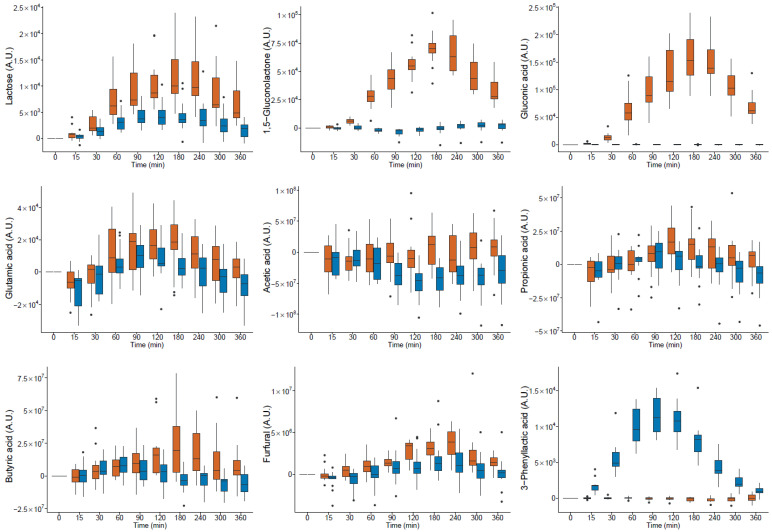

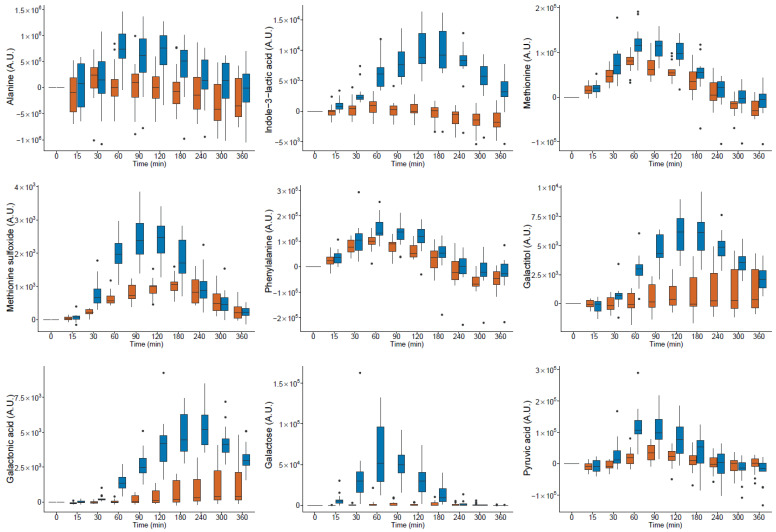
Kinetic of 18 postprandial active compounds in serum with a significant (*p* < 0.05) difference in the iAUC after the intake of 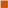 milk and 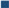 yogurt; A.U. arbitrary units (● outlier).

The postprandial iAUC of nine compounds was higher after milk intake than after yogurt intake; this difference was significant for four of them. The postprandial iAUC of 19 compounds was higher after yogurt intake than after milk intake, and was significant for 10 of them. The postprandial kinetics of the compounds in serum, with significant differences after milk versus yogurt intake, are shown in Figure 1.

Among the 30 postprandial active compounds in the serum, 22 were quantified in the dairy products (Table 1). Notably, 20 of them were significantly higher in yogurt than in milk. Only gluconic acid was significantly higher in milk, due to the addition of 1,5-gluconolactone.

In the urine pool after 6 hours, 25 of the 30 postprandial active compounds could be detected. The concentration of five compounds were higher after the intake of milk than after the intake of yogurt; this difference was significant for three compounds. The concentration of 20 compounds was higher after the yogurt intake than after the milk intake; this difference was significant for 12 compounds.

Among the 30 compounds with a postprandial effect after the intake of milk or yogurt, 29 were also detected in the serum after ingestion of HFM. Twenty-one of these compounds had a positive iAUC, which was significant for 10 of them. Eight compounds had negative iAUCs, five of which were significant. Only galactose could not be detected after the intake of HFM. Given that HFM was ingested in a higher caloric amount than the dairy products, the iAUC results of the individual metabolites were not statistically compared.

### 3.2. Overview of Nutrivolatilomic Results

After data deconvolution with Masshunter tools, a total of 1408 features in serum were integrated, with a peak height > 1500 units, with 669 features remaining following alignment. After FDR correction (*p* < 0.1), 160 features were confirmed as ‘postprandial active’ after milk or yogurt intake; 84 features (53%) showed a significant postprandial effect after milk intake, 40 features (25%) showed a significant postprandial effect after yogurt intake, and 36 features (23%) showed a significant postprandial effect after both milk and yogurt intake. Among the 160 features, 22 postprandial active compounds were identified at level 1 and were manually reintegrated (selected quantifier ion and defined retention indices) for final statistics (Table 2). Thirteen additional compounds (o-Xylene, pivalic acid, 2-propenoic acid, benzyl acetate, 2-butenoic acid, cis-2-methyl-2-butenoic acid, trans-2-methyl-2-butenoic acid, p-cresol, 4,5-dimethyl-3-hydroxy-2(5H)-furanone, propylbenzene, octanal, 1,2,4,5-tetramethylbenzene, and phenol) were already characterised in previous studies as postprandial active compounds and were thus present in the internal database. They were identified at level 1 and were also reintegrated for final statistics (Table 2). After manual reintegration, the iAUC of 30 compounds remained significantly different from zero (*p* < 0.05) after intake of milk or yogurt.

A comparison of the fasting results in serum before the intake of milk or yogurt did not show any significant difference between the two product groups for any of the 35 manually reintegrated compounds.

The largest group with 12 compounds was composed of acids and derivatives. We also identified 10 hydrocarbons, 3 aldehydes, 3 esters, 3 furans, 2 ketones, and 2 phenols.

The postprandial iAUC of 19 compounds was higher after milk intake than after yogurt intake; this difference was significant for three of them. The postprandial iAUCs of 14 of the compounds was higher after yogurt intake than after milk intake but the differences were not significant. Of note, acetic acid had a significantly more negative iAUC after milk intake than after yogurt intake. The postprandial kinetics of the compounds in serum, with significant differences after milk and yogurt intake, are shown in Figure 1.

In the 6 hour pooled urine samples, we detected 28 of the 35 postprandial active compounds. The concentration of 11 compounds were higher after the intake of milk than after the intake of yogurt; this difference was significant for two compounds. The concentration of 17 compounds were higher after the yogurt intake than after the milk intake, but these differences were not significant.

### 3.3. Kinetic Clustering of Postprandial Metabolites in Serum

**Figure 2:**
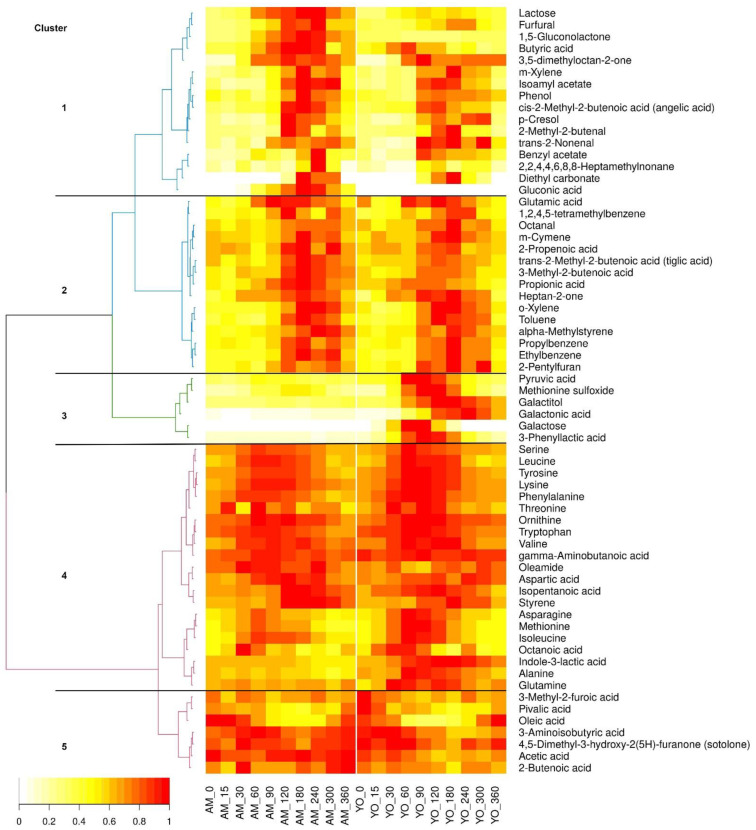
Heatmap with kinetics of 65 targeted postprandial active compounds in serum after consumption of milk (AM) and yogurt (YO) from 0 to 360 min.

The median kinetics of all 65 postprandial active compounds in serum are shown in the heatmap of Figure 2. The fasting profiles before milk and yogurt intake were similar, in agreement with the finding that no significant differences were found in the levels of these metabolites. Acetic acid, 2-butenoic acid, 3-aminobutyric acid, sotolone, oleic acid, pivalic acid, and 3-methyl-2-furoic acid grouped in cluster 5 and showed a negative iAUC over the 0–6 h time period after both milk and yogurt intake. Most amino acids and their derivatives grouped in cluster 4 and showed a similar kinetic with a relative maximum at 60 to 120 min after the intake of milk and yogurt. Galactose, galactonic acid, galactonate, methionine sulfoxide, pyruvic acid, and 3-phenyllactic acid grouped in cluster 3 and were more elevated in serum after the consumption of yogurt than after milk intake. Most hydrocarbons, octanal, heptan-2-one, glutamic acid, and four acids (2-propenoic acid, tiglic acid, 3-methyl-2-butenoic acid, and propionic acid) showed a similar kinetic with a relative maximum at 120 to 180 min after milk and yogurt intake (cluster 2). Lastly, lactose, furfural, 1,5-gluconolactone, butyric acid, and 3,5-dimethyloctan-2-one, m-xylene, isoamyl acetate, phenol, angelic acid, p-cresol, 2-mehyl-2-butenal, trans-2-nonenal, benzyl acetate 2,2,3,3,6,8,8,-heptamethylnonanone, diethyl carbonate, and gluconic acid grouped in cluster 1 and were more elevated in serum after milk intake than after yogurt intake.

### 3.4. Amino Acids and Their Derivatives

Due to the action of lactic acid bacteria during the production of yogurt, the concentration of most free amino acids is, on average, four times higher in yogurt than in milk [30]. Most of the proteinogenic amino acids measured showed a positive iAUC after the consumption of milk (11 of 15) and yogurt (13 of 15), in good agreement with previous work [17,31] (see Figure 1 for an illustration of the postprandial behaviour of alanine, glutamic acid, phenylalanine, and methionine). In the dairy products, the levels of 13 among these 15 proteinogenic amino acids were significantly higher in yogurt than in milk. Among these, three amino acids (alanine, methionine, and phenylalanine) had significantly higher iAUC in serum after yogurt intake compared to milk intake. Only glutamic acid had a significantly higher iAUC in serum after milk intake compared to yogurt intake. Six proteinogenic amino acids also showed significantly higher levels in the urine pools after yogurt ingestion compared to milk ingestion (alanine, asparagine, lysine, serine, tryptophan, and tyrosine). Alanine was consequently the only amino acid that showed significantly increased amounts associated with yogurt across all types of samples analysed (dairy products, serum, and urine). However, 9 proteinogenic amino acids out of the 15 measured, including alanine, also demonstrated a significant iAUC after HFM intake. By contrast, in our previous studies, the iAUCs of alanine in urine [16] and methionine and leucine in serum [32] were higher after cheese ingestion than after milk ingestion. Taken together, these results indicate that, although proteinogenic amino acids can obviously not be used as specific markers of dairy intake, the impact of fermentation of milk on the postprandial amino acid response supports the use of fermented dairy products to increase the short-term delivery of amino acids to the human organism. 

Three amino acid derivatives showed postprandial behaviour that was associated with the milk fermentation process. 3-Phenyllactic acid is an antimicrobial compound produced from the catabolism of phenylalanine [33]. 3-Phenyllactic acid was more elevated in yogurt than in milk, and the iAUC after yogurt intake was significantly higher in both serum and urine. The consumption of non-probiotic yogurt was recently demonstrated to produce more postprandial serum 3-phenyllactic acid than the consumption of non-acidified milk [18]. Similarly, postprandial blood 3-phenyllactic acid was higher after cheese ingestion than after milk ingestion [32]. Indole-3-lactic acid was also more elevated in yogurt than in milk, and the iAUC after yogurt intake was significantly higher in both serum and urine In this study, the same effect was already shown with LCMS [17]. Several intestinal bacteria, such as *Bacteroides*, *Clostridia*, and *E. coli*, can catabolise tryptophan to tryptamine and indole pyruvic acid, which are then converted to indole-3-acetic acid, indole propionic acid, and indole lactic acid [34]. Consequently, probiotics can be used to modulate the gut microbiota to selectively influence tryptophan metabolism and the production of these indoles [34]. Finally, the serum iAUC of methionine sulfoxide was significantly positive after the intake of milk and yogurt. Interestingly, however, the iAUC of methionine sulfoxide was significantly higher after the consumption of yogurt than after the consumption of milk (Figure 1), although it could not be detected in the dairy products or the pooled urine samples. In comparison, the iAUC of methionine was significantly higher after the consumption of yogurt (+153%) than after the consumption of milk. Methionine sulfoxide is an oxidation product of methionine that can be present in either a free or protein-bound form [35]. In calcium caseinate, up to 74% of the protein-bound methionine is present as methionine sulfoxide [36]. Methionine sulfoxide also appears in the blood of human subjects who ingest heat-treated whey proteins [37]. Thus, although methionine sulfoxide appears to be an interesting marker for the transformation of milk, this molecule can be produced in dairy products through heat treatment or fermentation. Of note, the iAUCs of 3-phenyllactic acid, indole-3-lactic acid, and methionine sulfoxide were not significantly positive after HFM intake. Gamma-amino butyric acid (GABA) is one of the most widely known neurotransmitter molecules increasingly documented in a range of neurological diseases [38]. In agreement with the known ability of lactic acid bacteria to produce GABA from glutamic acid [39], this molecule was significantly higher in yogurt than in milk (Table 1). However, although detected in serum and urine, a significant postprandial increase after milk or yogurt intake was not demonstrated in our study.

In summary, three proteinogenic amino acids (alanine, methionine, and phenylalanine) and three amino acid derivates (3-phenyllactic acid, indole-3-lactic acid, and methionine sulfoxide) showed an enhanced postprandial effect in serum that was associated with the milk fermentation process.

### 3.5. Carbohydrates and Their Derivatives

The milk used for this study contained 45 g/kg lactose and 0.05 g/kg galactose [21]. Due to the action of the yogurt culture, almost half of the lactose was degraded; 23.3 g/kg lactose and 1.8 g/kg galactose remained in the yogurt. Of note, the glucose content was below the detection limit in both dairy products. The iAUC of lactose was significantly positive after milk and yogurt intake but higher after milk intake compared to yogurt intake, both in the postprandial serum and pooled urine samples. Conversely, the iAUC of galactose, which was significantly positive after milk and yogurt intake, was higher after yogurt intake compared to milk intake both in the postprandial serum and urine pool.

Galactonic acid and galactitol are metabolites derived from galactose by the Leloir pathway in the liver: galactose dehydrogenase oxidises galactose to galactonate, whereas galactitol is formed by the action of aldose reductase [40]. The iAUCs of these molecules were consequently higher after yogurt intake compared to milk intake, both in postprandial blood and urine. A significant positive postprandial increase in lactose metabolites (lactose, galactose, galactonic acid, and galactitol) was not observed after intake of the HFM. Interestingly, postprandial galactitol and galactonate after a high dose of lactose appear to be good indicators of the inter-individual variability in lactase activity associated with genetic polymorphisms modulating lactase persistence [41]. 

The above results with lactose-derived metabolites are consistent with previous findings involving the consumption of non-probiotic yogurt and UHT milk [18], as well as pasteurised milk, and cheese [16,32]. Taken together, these studies support the profiling of these metabolites as potential markers of the intake of products containing lactose (including fermented and non-fermented dairy products, composite dishes, and drugs).

Interestingly, a significant postprandial pyruvate response was observed after yogurt intake in serum and urine but not after milk intake. During yogurt production, pyruvate is produced from glucose and subsequently transformed into lactate by the action of lactic acid bacteria [42]. Although pyruvate was not detected by our GC-MS method in dairy products, the presence of this molecule has been reported in yogurt using NMR spectroscopy [43,44]. The postprandial pyruvate observed after yogurt intake in our study likely originated from the fermented product. Notably, although pyruvic acid was found in fermented cocoa pulp [45], a postprandial pyruvate response observed after the acute intake of flavan-3-ol-enriched dark chocolate was postulated to be derived from an endogenous production [46]. Additionally, the intake of tea, which is usually not fermented [47], resulted in a postprandial pyruvate production in humans [48]. Following the postprandial fate of the axis glucose-pyruvate-lactate in human intervention studies investigating the impact of food fermentation on human energy metabolism [49] therefore appears to be an interesting research avenue.

In this study, milk was acidified with 1,5-gluconolactone to allow for a crossover intervention study comparing two dairy products (i.e., milk and probiotic yogurt) with similar textures. 1,5-Gluconolactone and its metabolite gluconic acid consequently appeared in the postprandial serum and urine after milk intake but only at a very low level after yogurt intake.

### 3.6. Organic Acids and Oleamide

Fatty acids (C4–C22) are mostly present in cow’s milk as triglycerides and their composition depends mainly on the feed of the cows [50,51]. Free short-, medium-, and long-chain carboxylic acids are formed during yogurt production and many of them increase during storage [52,53,54,55]. Acute effects of fat in meals on postprandial non-esterified fatty acids (NEFA) typically show a sharp decrease in NEFA responses up to 120 min, with NEFA returning to baseline levels after 5 to 6 h [56].

Oleic acid, among other fatty acids, showed a postprandial decrease of up to 120 min and is a reliable indicator of normally regulated metabolism [57]. Total oleic acid is present in cow milk at a concentration of 11–16 g/100 g fat, depending on the feed [58]. In our study, the iAUC of oleic acid was significantly negative after the intake of both dairy products and the HFM, confirming the typical expected profile for this fat. Although oleic acid was present at significantly higher concentrations in yogurt than in milk, no difference in the postprandial response was measured in the blood or urine.

The three short-chain carboxylic acids (SCCAs), acetic acid, propionic acid, and butyric acid, are important molecules derived by the gut microbiota from the metabolism of food, particularly dietary fibres. These molecules are involved in regulating the cell cycle, neurobiological signalling, cholesterol and bile acid metabolism, immune responses, and responses to antioxidants [59]. Acetic acid has been indicated to be the SCCA with the highest concentration in the serum of healthy subjects [60]. Interestingly, the postprandial levels of acetic acid and propionic acid decreased and increased, respectively, after the ingestion of a hamburger [61,62]. Although dietary fibres represent the main nutritional strategy to increase the production of SCCAs in humans [63], attempts have been undertaken to modulate the production of these molecules through the ingestion of probiotics [64]. Taken together, these findings raise the question of the impact of milk transformation to (probiotic) yogurt in our study on postprandial SCCAs. In agreement with the findings for the hamburger challenge, we found a postprandial decrease in acetic acid as well as an increase in postprandial propionic acid after intake of the dairy products and the HFM. The iAUC of butyric acid was also significantly positive after milk intake, in line with the finding that butyric acid is present in milk at a concentration of 3.0–3.3 g/100 g of fat [58]. Interestingly, the negative iAUC of acetic acid was significantly more pronounced after yogurt intake compared to milk intake, whereas the positive iAUCs of butyric and propionic acids were significantly higher after the intake of milk than after the intake of yogurt (Figure 1), suggesting the effect of milk fermentation to yogurt. Given the addition of *Lactobacillus rhamnosus* Gorbach-Goldin (LGG) to the yogurt, this could also suggest the potential of this probiotic to modulate the postprandial SCCA response. Of note, however, 1,5-gluconolactone was used for the acidification of milk, which resulted in the production of gluconic acid. In the cecal digesta of pigs, gluconic acid stimulates butyric acid production [65]. Dietary sodium gluconate also promotes the production of butyric acid in the large intestines of rats [66]. Taken together, our results indicate that SCCAs can be modulated postprandially via the interplay of a complex array of factors that include fermentation, pre- and/or probiotic activities, and gut microbiota.

A range of other organic acids exhibited significant postprandial changes after the intake of milk (octanoic acid), yogurt (2,2-dimethylpropionic acid (pivalic acid)), HFM (3-methyl-2-butenoic acid (senecioic acid)), yogurt and the HFM (3-methyl-2-furoic acid), or both dairy products and HFM (cis-2-methyl-2-butenoic acid (angelic acid)). Associations of these organic acids with diverse foods have been reported. Pivalic acid has been detected but not quantified in different animal foods and could be a potential biomarker for their consumption [67]. 2-Butenoic acid was identified in the fruit pulp of melon [68]. 3-Methyl-2-furoic acid was detected in Chukrasia velutina leaves [69]. Cis-2-methyl-2-butenoic acid (angelic acid) has been found in fats and oils [70]. 3-Methyl-2-butenoic acid (senecioic acid) has been found in various foods [71] as trans-2-methyl-2-butenoic acid (tiglic acid) [72]. Finally, octanoic acid is present in milk at a concentration of 1.2–1.5 g/100 g of fat [58].

### 3.7. Aldehydes

Although the iAUC of 2-methyl-2-butenal (tiglic aldehyde), octanal, and trans-2-nonenal were significantly positive in serum after the intake of dairy products, (except for 2-methyl-2-butenal after milk intake), none of the iAUC differed when milk and yogurt intake were compared, either in serum or urine. Tiglic aldehyde has been found in plant and animal foods [72]. Octanal has been found in many fruits and vegetables; it has also been detected in several cheese varieties [73] and is known as a biomarker of lipid peroxidation [74]. Trans-2-nonenal is found in many plant foods; its presence has also been suggested but not identified at level 1 (ID: PM4) in plasma after the intake of a soy drink [15]. 

### 3.8. Esters

The iAUCs of diethyl carbonate, isoamyl acetate, and benzyl acetate were significantly positive after the intake of milk and the HFM but only for isoamyl acetate after the intake of yogurt. The iAUCs for these molecules were not significantly different after milk and yogurt intake, except for a higher iAUC of benzyl acetate in the urine pool after milk intake compared to yogurt intake. Benzyl acetate is a fermentation product of grapes during their transformation to wine [75]. 

### 3.9. Furans

The iAUC of 2-pentylfuran in postprandial serum was significantly positive after the intake of both dairy products and the HFM. The iAUC of furfural in postprandial serum was significantly positive after the intake of milk and HFM, as well as significantly higher after milk intake when compared to yogurt intake (Figure 1). Both furans were detected in urine samples, and the iAUC of furfural in urine was significantly higher after milk intake than after yogurt intake. 2-Pentylfuran is present in many foods [76,77]. Furfural has been found in coffee, calamus, matsutake mushroom, pumpkin, malt, peated malt, Bourbon vanilla, Lamb’s lettuce, pimento leaf, various fruits, and Chinese quince, and is a common constituent of essential oils [78]. 

### 3.10. Ketons

The iAUCs of heptan-2-one and 3,5-dimethyloctan-2-one were significantly positive after the intake of both dairy products. The iAUC of heptan-2-one was also significantly positive after HFM intake. Interestingly, 3,5-dimethyloctan-2-one was detected only in trace levels in serum after HFM intake, making it an interesting candidate marker of dairy intake, as it confirms the hypothesis proposed in a previous study in which both increased in blood samples taken after intake of milk and cheese [15]. Neither ketone was detected in the postprandial urine pools. Heptan-2-one has been found in different foods and herbs, including cow milk, while 3,5-dimethyloctan-2-one has not been detected in in milk, cheese and soy drink to date [15].

### 3.11. Hydrocarbons

The iAUCs of 10 hydrocarbons (toluene, ethylbenzene, m-xylene, o-xylene, propylbenzene, 2,2,4,4,6,8,8-heptamethylnonane (isocetane), styrene, m-cymene, alpha-methylstyrene, and 1,2,4,5-tetramethylbenzene (durene)) were significantly positive after the intake of both dairy products as well as after HFM intake. With the exception of isocetane in the urine samples, each of these hydrocarbons was detected in the fasting sera and in urine. Toluene is found in many different foods, such as black walnuts, rosemary, kohlrabi, cow milk, and cheese [73]. The highest amounts of ethylbenzene are found in black walnuts and safflowers, but it has also been detected in several vegetables [79]. m-Xylene is found in the highest amounts in safflowers but is also present in black walnuts and parsley [79]. o-Xylene has been found in the highest amounts in black walnuts but is also present in fruits and vegetables [79]. Propylbenzene has been quantified in human urine and plasma [80]. Styrene has been detected in dairy, meat, poultry, vegetables, soups, cereals, fruits, and many other foods [81]. m-Cymene was detected in sweet basils, blackcurrants, and fruits [79]. Alpha-methylstyrene has been found in serum samples of patients with different stages of COVID-19 infection [82]. Durene has been detected in sweet cherries [83]. 

However, their presence in foods and human fluids raises the question of the evaluation of chemical contaminants along the food production chain [84,85], particularly in the context of the dramatically increasing sensitivity of analytical technologies [86].

### 3.12. Phenols

The iAUCs of phenol and p-cresol were significantly positive after the intake of both dairy products, whereas only the iAUC of p-cresol was significantly positive after HFM intake. Both molecules were detected in the fasting sera and urine samples. Phenol has been detected in several fruits and vegetables [79] and quantified in the human urine metabolome [87,88]. p-Cresol is an end product of aromatic amino acid metabolism and increases in urine with nutritional protein intake. It has also been quantified in the human urine metabolome [87]. 

### 3.13. Use of Different Metabolomics Platforms for Biomarker Discovery

We previously published the results from an analysis of the metabolome of the serum samples of this human intervention study using an LC-MS platform [17]. Using this platform, 20 metabolites were identified that were postprandially discriminant for the intake of milk and yogurt. Among these metabolites, nine were identified again using the GC-MS platform with derivatisation. The discriminating postprandial response of four of them was statistically confirmed by increased levels after yogurt intake for phenylalanine and indole-3-lactic acid and after milk intake for gluconic acid and 1,5-gluconolactone. For five of these compounds, all amino acids (asparagine, lysine, threonine, tyrosine, and tryptophan), the direction of change (i.e., increased after yogurt intake) was confirmed, although the effect did not reach statistical significance in this study. The use of different metabolomics platforms to characterise study samples also allows the acquisition of complementary sets of metabolites of interest (e.g., metabolites detected only by specific platforms). In this context, our datasets revealed new discriminating metabolites of dairy intake but also point to specific limitations in the precision of the quantification of the measured features, highlighting the importance of more precise quantitative methods for the validation of key markers of intake in subsequent studies [14].

## 4. Conclusions

This study has shown the effects of the acute consumption of acidified milk and probiotic yogurt in young and healthy men. A non-dairy HFM was used for comparison of the postprandial response. Two independent GC methods were used for the study, the volatile extraction method allowing to expand the number of molecules measured by GC-MS beyond the classical method with derivatisation.

Our study identified a range of metabolites whose postprandial response was specifically modified by the transformation of milk to yogurt:

Lactose and its bacterial/intestinal (galactose) and liver (galactitol, galactonate) metabolites are postprandial indicators of milk fermentation that should be further investigated, particularly in observational studies, as markers of dairy intake, and more specifically as markers of the intake of lactose-containing products (dairy products, composite dishes, drugs). When combined, these markers could provide information on the fermentation status of ingested dairy products. Their levels in blood and urine have previously been shown to depend on the genetic polymorphisms that determine lactase persistence [41]. Taken together, these properties render the use of lactose and its metabolites complex but promising biomarkers in association with dairy intake.

Indole-3-lactic acid and 3-phenyllactic acid are produced by fermentation of milk, and their postprandial presence in blood and urine samples in this study was characterised by a strong signal not seen after milk or HFM intake, making them attractive candidates as markers, not of yogurt or even cheese [16,17,32], but more likely of fermented foods. These molecules deserve further investigation as BFIs. The combination of several compounds in a multi-marker analysis has led to an improvement in the prediction of milk and cheese consumption [89]. Recently, the web application multiMarker was launched to model and predict food intake using multimarker biomarkers. The software is an advance in the field of biomarkers of dietary intake by providing a novel tool to continuously quantify food intake and assess the associated uncertainty using multiple biomarkers [90].

We previously showed that the volatile compound 3,5-dimethyloctan-2-one appears postprandially in blood after the intake of milk and cheese but not after a soy-based drink [15]. Our current study characterised the ingestion of milk and yogurt, but not of the non-dairy HFM composed of bread, egg, salami, and palm oil, by a clear postprandial 3,5-dimethyloctan-2-one signal, making it an interesting candidate marker for dairy products.

Free amino acids derived from dairy products are generally increased postprandially when yogurt is compared to milk intake. This effect, although of moderate magnitude, has previously been reported for cheese intake [16]. Taken together, these studies provide sound evidence for the use of fermentation to improve the acute delivery of proteinogenic amino acids to humans.

In total, more than 600 features showed a significant postprandial effect after milk or yogurt consumption using the two GC methods. Of these, only 65 compounds were identified at levels 1 or 2. Therefore, there is still a large potential for the discovery and characterisation of interesting biomarkers associated with the intake and/or effect of dairy products. The correct identification of these compounds is still a time-consuming bottleneck that could be alleviated through the improved availability of reference materials. However, many compounds in sera after HFM intake had a significant positive postprandial response (Figure S1), precludes their use as individual markers of the intake of specific foods, such as dairy products.

## Figures and Tables

**Table 1:** Characteristics of postprandial active metabolites derived from the GC-MS analysis with derivatisation after the intake of milk (AM), yogurt (YO), and high-fat meal (HFM) by healthy men.

Compound	HMDB Database Entry	RT	RI	Quantifier Ion	Qualifier Ion	Level ID ^b^	Postprandial Response (iAUC) in Serum after Milk Intake ^c^			Postprandial Response (iAUC) in Serum after Yogurt Intake ^d^			Product with Higher Post-Prandial Response (iAUC) in Serum ^f^	Product with Higher Content in 6 h Urine Pool ^g^	Product with Higher Content ^h^	Postprandial Response (iAUC) in Serum after HFM Intake ^i^
		(min)	( )	(m/z) ^a^	(m/z) ^a^		Median	SD		Median	SD					
Amino acids and derivatives																
Alanine	HMDB0000161	14.87	1094	116	190	1	−4.58 × 10^7^	1.20 × 10^8^		1.42 × 10^8^	1.24 × 10^8^	*	YO *	YO *	YO *	pos *
Asparagine	HMDB0000168	27.46	1658	116	231	1	2.92 × 10^6^	4.48 × 10^6^	*	6.09 × 10^6^	4.41 × 10^6^	*	YO	YO *	YO *	pos *
Aspartic acid	HMDB0000191	25.13	1508	232	218	1	2.52 × 10^6^	3.08 × 10^6^	*	2.35 × 10^6^	2.14 × 10^6^	*	AM	YO	YO*	pos
Glutamic acid	HMDB0000148	26.74	1607	246	128	1	4.00 × 10^6^	4.51 × 10^6^	*	9.36 × 10^5^	3.36 × 10^6^		AM *	ND	YO	pos
Glutamine	HMDB0000641	28.80	1764	156	245	2	6.02 × 10^6^	1.75 × 10^7^		1.88 × 10^7^	3.05 × 10^7^	*	YO	ND	ND	pos
Isoleucine	HMDB0000172	20.27	1286	158	218	1	8.20 × 10^7^	2.00 × 10^7^	*	7.76 × 10^7^	3.02 × 10^7^	*	AM	YO	YO*	pos *
Leucine	HMDB0000687	19.69	1265	158	232	1	1.20 × 10^8^	3.98 × 10^7^	*	1.15 × 10^8^	5.41 × 10^7^	*	AM	YO	YO*	pos *
Lysine	HMDB0000182	30.64	1914	174	317	1	3.51 × 10^7^	1.98 × 10^7^	*	4.46 × 10^7^	1.58 × 10^7^	*	YO	YO*	YO*	pos *
Methionine	HMDB0000696	25.21	1513	176	128	1	7.16 × 10^6^	5.51 × 10^6^	*	1.81 × 10^7^	8.09 × 10^6^	*	YO *	YO	YO*	pos *
Phenylalanine	HMDB0000159	26.97	1623	218	192	1	6.44 × 10^6^	8.97 × 10^6^	*	1.93 × 10^7^	1.65 × 10^7^	*	YO *	YO	YO*	pos *
Serine	HMDB0062263	21.88	1351	204	218	1	2.42 × 10^7^	1.88 × 10^7^	*	2.72 × 10^7^	3.16 × 10^7^	*	YO	YO*	YO*	pos *
Threonine	HMDB0000167	20.31	1288	117	130	1	1.38 × 10^6^	4.84 × 10^6^		2.60 × 10^6^	2.14 × 10^6^	*	YO	YO	YO*	pos
Tryptophan	HMDB0000929	33.52	2215	202	203	2	3.65 × 10^6^	2.79 × 10^7^		2.03 × 10^7^	3.76 × 10^7^		YO	YO*	YO*	pos
Tyrosine	HMDB0000158	30.82	1932	218	280	1	4.35 × 10^7^	2.31 × 10^7^	*	6.12 × 10^7^	2.27 × 10^7^	*	YO	YO*	YO*	pos
Valine	HMDB0000883	18.10	1209	144	218	1	1.17 × 10^8^	5.87 × 10^7^	*	1.21 × 10^8^	7.53 × 10^7^	*	YO	AM	YO*	pos *
3-Aminoisobutyric acid (BAIBA)	HMDB0003911	24.17	1455	248	304	1	−3.62 × 10^4^	2.07 × 10^5^		−1.14 × 10^5^	2.00 × 10^5^	*	-YO	AM	ND	pos
3-Phenyllactic acid	HMDB0000779	26.33	1579	193	220	1	−6.67 × 10^3^	9.32 × 10^4^		2.18 × 10^6^	2.46 × 10^5^	*	YO *	YO*	YO*	neg
gamma-Amino-butanoic acid (GABA)	HMDB0000112	25.39	1524	174	304	1	7.76 × 10^4^	5.71 × 10^5^		6.27 × 10^4^	1.20 × 10^6^		AM	YO	YO*	pos
Indole-3-lactic acid	HMDB0000671	33.14	2172	202	203	1	−1.62 × 10^5^	4.69 × 10^5^		2.38 × 10^6^	9.47 × 10^5^	*	YO *	YO*	YO*	neg *
Methionine sulfoxide	HMDB0002005	28.95	1775	128	174	1	2.22 × 10^5^	6.61 × 10^4^	*	4.38 × 10^5^	8.02 × 10^4^	*	YO *	ND	ND	pos
Ornithine	HMDB0000214	29.34	1806	142	174	1	2.32 × 10^7^	1.41 × 10^7^	*	1.90 × 10^7^	1.21 × 10^7^	*	AM	ND	ND	pos *
Lipid compounds																
Oleamide	HMDB0002117	35.28	2415	144	338	1	2.03 × 10^6^	4.79 × 10^6^		2.57 × 10^6^	5.70 × 10^6^		YO	ND	ND	neg
Oleic acid	HMDB0000207	33.50	2213	339	117	1	−3.91 × 10^7^	3.35 × 10^7^	*	−2.44 × 10^7^	3.00 × 10^7^	*	-AM	YO	YO *	neg *
Carbohydrates and derivatives																
Lactose	HMDB0041627	37.90	2664	480	451	1	2.65 × 10^6^	1.38 × 10^6^	*	9.57 × 10^5^	6.75 × 10^5^	*	AM *	AM *	AM *^,e^	neg
Galactose	HMDB0000143	30.51	1906	319	205	1	7.67 × 10^4^	6.13 × 10^5^	*	6.12 × 10^6^	2.38 × 10^6^	*	YO *	YO *	YO *	ND
Galactitol	HMDB0000107	30.70	1924	217	307	1	1.82 × 10^4^	5.13 × 10^5^		1.49 × 10^6^	3.22 × 10^5^	*	YO *	YO *	ND	pos
Galactonic acid	HMDB0000565	31.28	1978	292	333	1	6.15 × 10^4^	2.81 × 10^5^	*	1.22 × 10^6^	3.62 × 10^5^	*	YO *	YO *	YO *	neg *
Pyruvic acid	HMDB0000243	13.33	1038	174	115	1	3.21 × 10^6^	7.33 × 10^6^		1.22 × 10^7^	1.26 × 10^7^	*	YO *	YO *	ND	pos
GDL and derivatives																
1,5-Glucono-lactone	HMDB0000150	30.11	1870	229	189	1	1.70 × 10^7^	2.90 × 10^6^	*	2.26 × 10^5^	1.03 × 10^6^		AM *	AM *	ND	neg *
Gluconic acid	HMDB0000625	31.33	1990	292	333	1	3.87 × 10^7^	6.99 × 10^6^	*	−7.88 × 10^3^	1.13 × 10^5^		AM *	AM *	AM *	neg *

RT, retention time; RI, retention index; ND, not detected; AM, milk; YO, yogurt; HFM, high-fat meal; ^a^ quantifier ion and qualifier ion retrieved from deconvoluted data; ^b^ Identification level 1: Compounds were identified by comparison to a pure reference (injection); level 2: Compounds were identified based on a spectral database; ^c^ SD standard deviation after milk intake; *, *p* < 0.05 of Wilcoxon test, if iAUC is different from 0; ^d^ SD standard deviation after yogurt intake; *, *p* < 0.05 of Wilcoxon test, if iAUC is different from 0; ^e^ The lactose content (mean ± SEM) in milk was 4.5 ± 0.01 g/100 g and 2.3 ± 0.04 g/100 g in yogurt; ^f,^*, *p* < 0.05 of paired Wilcoxon test, if the postprandial response (iAUC) in serum is different after milk and yogurt intake; ^g,^*, *p* < 0.05 of paired Wilcoxon test, if the content in the 6 h urine pool is different after milk and yogurt intake; -AM/-YO: negative iAUC after milk/yogurt intake; ^h,^*, *p* < 0.05 of Wilcoxon test, if the content in the product is significantly different between milk and yogurt; ^i^ pos, positive iAUC; neg, negative iAUC; *, *p* < 0.05 of Wilcoxon test, if the postprandial response (iAUC) in serum is significantly different from 0.

**Table 2:** Characteristics of postprandial active metabolites derived from the volatilomics analysis after the intake of milk (AM), yogurt (YO), and high-fat meal (HFM) by healthy men.

Compound	HMDB Database Entry	RT	RI	Quantifier Ion	Postprandial Response (iAUC) in Serum after Milk Intake			Postprandial Response (iAUC) in Serum after Yogurt Intake			Product with Higher Post-Prandial Response (iAUC) in Serum	Product with Higher Content in 6 h Urine Pool	Postprandial Response (iAUC) in Serum after HFM Intake
		(min)	( )	(m/z)	Median	SD		Median	SD				
Carboxylic acids													
Acetic acid	HMDB0000042	29.10	1476	60	−1.9 × 10^7^	8.5 × 10^9^		−1.2 × 10^10^	7.3 × 10^9^	*	-YO *	YO	neg*
Propionic acid	HMDB0000237	32.64	1560	74	2.8 × 10^9^	4.2 × 10^9^	*	2.6 × 10^8^	4.2 × 10^9^		AM *	YO	pos *
2,2-Dimethyl-propionic acid (pivalic acid)	HMDB0041992	34.16	1596	69	−2.3 × 10^7^	8.4 × 10^7^		−6.2 × 10^7^	8.2 × 10^7^	*	-YO	ND	pos
Butyric acid	HMDB0000039	36.38	1652	73	3.4 × 10^9^	5.3 × 10^9^	*	1.2 × 10^8^	2.6 × 10^9^		AM *	AM	pos
2-Propenoic acid	HMDB0031647	36.63	1658	72	4.0 × 10^8^	1.2 × 10^9^		3.0 × 10^8^	4.8 × 10^8^		AM	AM	neg
Isopentanoic acid	HMDB0000718	37.98	1692	87	4.5 × 10^9^	8.2 × 10^9^		3.0 × 10^9^	6.3 × 10^9^		AM	YO	pos
2-Butenoic acid	HMDB0010720	42.02	1800	86	2.3 × 10^8^	1.1 × 10^9^		−2.8 × 10^8^	7.8 × 10^8^	*	AM	ND	neg
cis-2-Methyl-2-butenoic acid (angelic acid)	HMDB0029608	42.26	1806	100	1.0 × 10^8^	1.6 × 10^8^	*	1.1 × 10^8^	8.7 × 10^7^	*	YO	AM	pos *
3-Methyl-2-butenoic acid (senecioic acid)	HMDB0000509	42.80	1821	100	4.1 × 10^8^	7.6 × 10^8^		3.2 × 10^8^	7.6 × 10^8^		AM	YO	pos *
trans-2-Methyl-2-but-enoic acid (tiglic acid)	HMDB0001470	44.54	1869	100	8.9 × 10^8^	1.7 × 10^9^		6.1 × 10^8^	1.2 × 10^9^		AM	YO	pos
Octanoic acid	HMDB0000482	51.83	2083	115	1.1 × 10^9^	1.1 × 10^9^	*	9.6 × 10^8^	2.0 × 10^9^		AM	AM	neg
3-Methyl-2-furoic acid	NA	66.70	2569	126	−3.2 × 10^8^	1.9 × 10^9^		−1.5 × 10^9^	1.8 × 10^9^	*	AM	YO	pos *
Aldehydes													
2-Methyl-2-butenal (tiglic aldehyde)	HMDB0031512	12.74	1124	84	6.7 × 10^8^	6.7 × 10^8^		7.0 × 10^8^	4.7 × 10^8^	*	YO	YO	pos
Octanal	HMDB0001140	21.88	1316	41	3.0 × 10^7^	6.5 × 10^7^	*	5.4 × 10^7^	5.9 × 10^7^	*	YO	YO	pos
trans-2-Nonenal	HMDB0255708	32.82	1564	81	1.5 × 10^8^	1.6 × 10^8^	*	1.9 × 10^8^	1.7 × 10^8^	*	YO	YO	pos *
Esters													
Diethyl carbonate	HMDB0059844	13.27	1135	91	1.4 × 10^7^	1.0 × 10^7^	*	1.5 × 10^7^	1.5 × 10^7^		YO	ND	pos *
Isoamyl acetate	HMDB0031528	13.85	1147	43	5.0 × 10^7^	5.1 × 10^7^	*	4.3 × 10^7^	6.8 × 10^7^	*	AM	ND	pos *
Benzyl acetate	HMDB0031310	40.62	1762	108	7.5 × 10^7^	9.4 × 10^7^	*	6.7 × 10^7^	1.6 × 10^8^		AM	AM *	pos *
Furans													
2-Pentylfuran	HMDB0013824	19.01	1255	81	2.0 × 10^8^	3.0 × 10^8^	*	3.5 × 10^8^	2.0 × 10^8^	*	YO	YO	pos *
2-Furancarboxal-dehyde (furfural)	HMDB0032914	30.16	1500	96	7.1 × 10^8^	2.8 × 10^8^	*	3.3 × 10^8^	4.6 × 10^8^		AM *	AM *	pos *
4,5-Dimethyl-3-hydroxy-2(5H)-furanone (sotolone)	HMDB0031306	56.66	2238	128	1.1 × 10^8^	2.1 × 10^9^		−8.8 × 10^8^	4.8 × 10^9^		AM	AM	pos
Ketones													
Heptan-2-one	HMDB0003671	16.90	1211	114	8.2 × 10^6^	5.8 × 10^6^	*	1.1 × 10^7^	5.4 × 10^6^	*	YO	ND	pos *
3,5-dimethyloctan-2-one	NA	24.51	1374	72	4.2 × 10^7^	2.3 × 10^7^	*	4.6 × 10^7^	2.1 × 10^7^	*	YO	ND	neg
Hydrocarbons													
Toluene	HMDB0034168	10.15	1067	91	4.6 × 10^9^	4.0 × 10^9^	*	4.9 × 10^9^	3.4 × 10^9^	*	YO	YO	pos *
Ethylbenzene	HMDB0059905	13.93	1149	91	2.7 × 10^8^	3.2 × 10^8^	*	3.1 × 10^8^	1.8 × 10^8^	*	YO	YO	pos *
m-Xylene	HMDB0059810	14.62	1163	91	8.1 × 10^8^	1.2 × 10^9^	*	8.7 × 10^8^	4.2 × 10^8^	*	YO	YO	pos *
o-Xylene	HMDB0059851	16.90	1211	91	3.6 × 10^8^	3.3 × 10^8^	*	4.1 × 10^8^	2.8 × 10^8^	*	YO	YO	pos *
Propylbenzene	HMDB0059877	17.94	1233	91	1.2 × 10^8^	1.3 × 10^8^	*	1.6 × 10^8^	7.8 × 10^7^	*	YO	AM	pos *
2,2,4,4,6,8,8-Heptamethylnon-ane (isocetane)	NA	20.08	1278	57	1.9 × 10^8^	1.9 × 10^8^	*	9.0 × 10^7^	2.0 × 10^8^	*	AM	ND	pos *
Styrene	HMDB0034240	20.50	1287	104	8.7 × 10^8^	8.7 × 10^8^	*	7.5 × 10^8^	9.0 × 10^8^	*	AM	AM	pos *
m-Cymene	HMDB0037051	20.86	1294	119	3.4 × 10^8^	3.0 × 10^8^	*	3.1 × 10^8^	3.0 × 10^8^	*	AM	YO	pos *
alpha-Methyl-styrene	HMDB0059899	23.87	1360	103	1.4 × 10^8^	9.6 × 10^7^	*	1.0 × 10^8^	1.4 × 10^8^	*	AM	YO	pos *
1,2,4,5-tetra-methyl-benzene (durene)	HMDB0244147	28.53	1463	119	1.9 × 10^8^	3.0 × 10^8^	*	2.5 × 10^8^	3.4 × 10^8^	*	YO	YO	pos *
Phenols													
Phenol	HMDB0000228	50.57	2044	94	4.5 × 10^9^	4.6 × 10^9^	*	3.8 × 10^9^	1.1 × 10^10^	*	AM	AM	pos
p-Cresol	HMDB0001858	53.37	2131	77	7.3 × 10^8^	2.0 × 10^9^	*	5.2 × 10^8^	1.5 × 10^9^	*	AM	AM	pos *

Legend: see Table 1. *, *p* < 0.05; NA: not available. -AM/-YO: higher negative iAUC after milk/yogurt intake.

## Data Availability

The data presented in this study are available on request from the corresponding author.

## Supplementary Materials

The following supporting information can be downloaded at: https://www.mdpi.com/article/10.3390/nu14224794/s1, Figure S1: Postprandial response (iAUC) boxplots of 65 postprandial active compounds in serum after consumption of milk (AM), yogurt (YO), and high fat meal (HFM).
