# Supplementary material for: Serum and Urine Metabolites in Healthy Men after Consumption of Acidified Milk and Yogurt
Source: Nutrients. 2022 Nov 12;14(22):4794. doi: 10.3390/nu14224794 (PMC9698558; doi:10.3390/nu14224794)
Supplement: Supplementary file 1 [file nutrients-14-04794-s001.zip › Supplementary Materials.pdf]

Serum and urine metabolites in healthy men after consumption of acidified milk and yogurt

Ueli Bütikofer, René Badertscher, Carola Blaser-Freiburghaus, Pascal Fuchsmann, Mireille Tena Stern, Philipp A. Kuert, Grégory Pimentel, Kathryn Jane Burton-Pimentel, Nathalie Vionnet and Guy Vergères

Supplementary Materials:

Figure S1: Postprandial response (iAUC) boxplots of 65 postprandial active compounds in serum after consumption of milk (AM), yogurt (YO), and high fat meal (HFM).

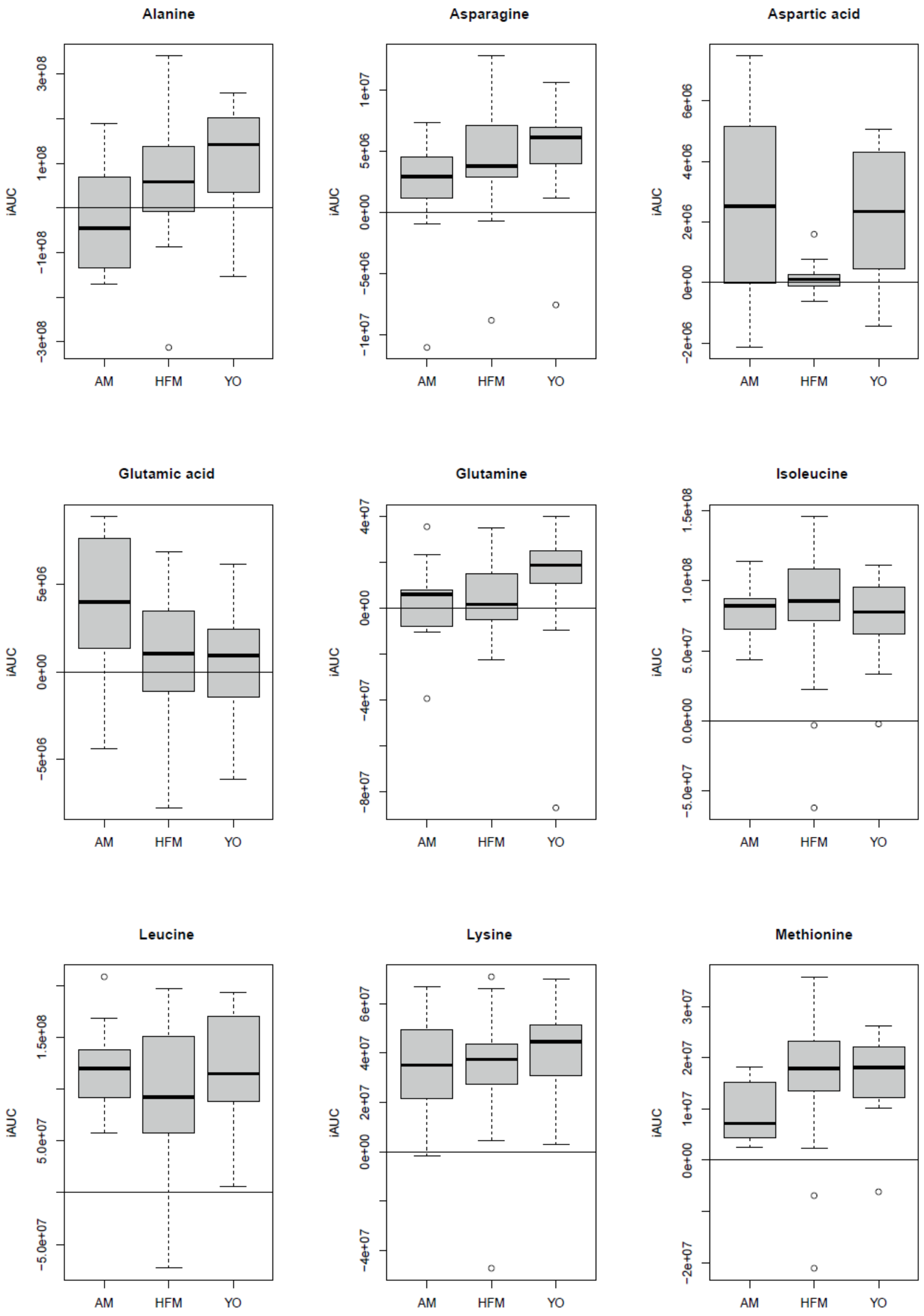

Alanine
iAUC
3e+08
1e+08
−1e+08
−3e+08
AM
HFM
YO
Asparagine
iAUC
1e+07
5e+06
0e+00
−5e+06
−1e+07
AM
HFM
YO
Aspartic acid
iAUC
6e+06
4e+06
2e+06
0e+00
−2e+06
AM
HFM
YO
Glutamic acid
iAUC
5e+06
0e+00
−5e+06
AM
HFM
YO
Glutamine
iAUC
4e+07
0e+00
−4e+07
−8e+07
AM
HFM
YO
Isoleucine
iAUC
1.5e+08
1.0e+08
5.0e+07
0.0e+00
−5.0e+07
AM
HFM
YO
Leucine
iAUC
1.5e+08
5.0e+07
−5.0e+07
AM
HFM
YO
Lysine
iAUC
6e+07
4e+07
2e+07
0e+00
−4e+07
AM
HFM
YO
Methionine
iAUC
3e+07
2e+07
1e+07
0e+00
−2e+07
AM
HFM
YO

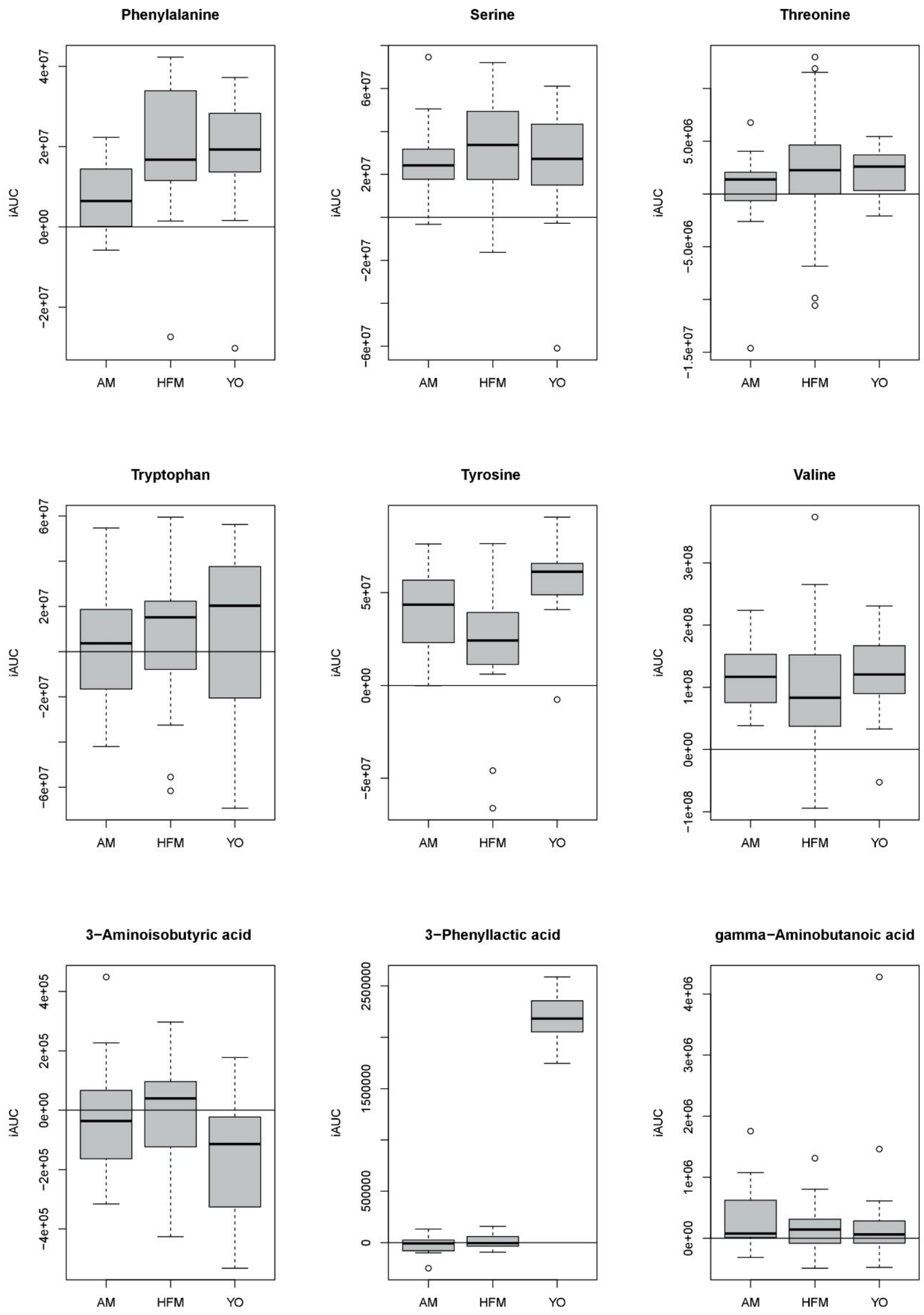
Phenylalanine
Serine
Threonine
Tryptophan
Tyrosine
Valine
3−Aminoisobutyric acid
3−Phenyllactic acid
gamma−Aminobutanoic acid
iAUC
AM
HFM
YO
4e+07
2e+07
0e+00
−2e+07
6e+07
−6e+07
5.0e+06
−5.0e+06
−1.5e+07
5e+07
−5e+07
3e+08
2e+08
1e+08
0e+00
−1e+08
4e+05
2e+05
−2e+05
−4e+05
2500000
1500000
500000
0
4e+06
3e+06
2e+06
1e+06
0e+00

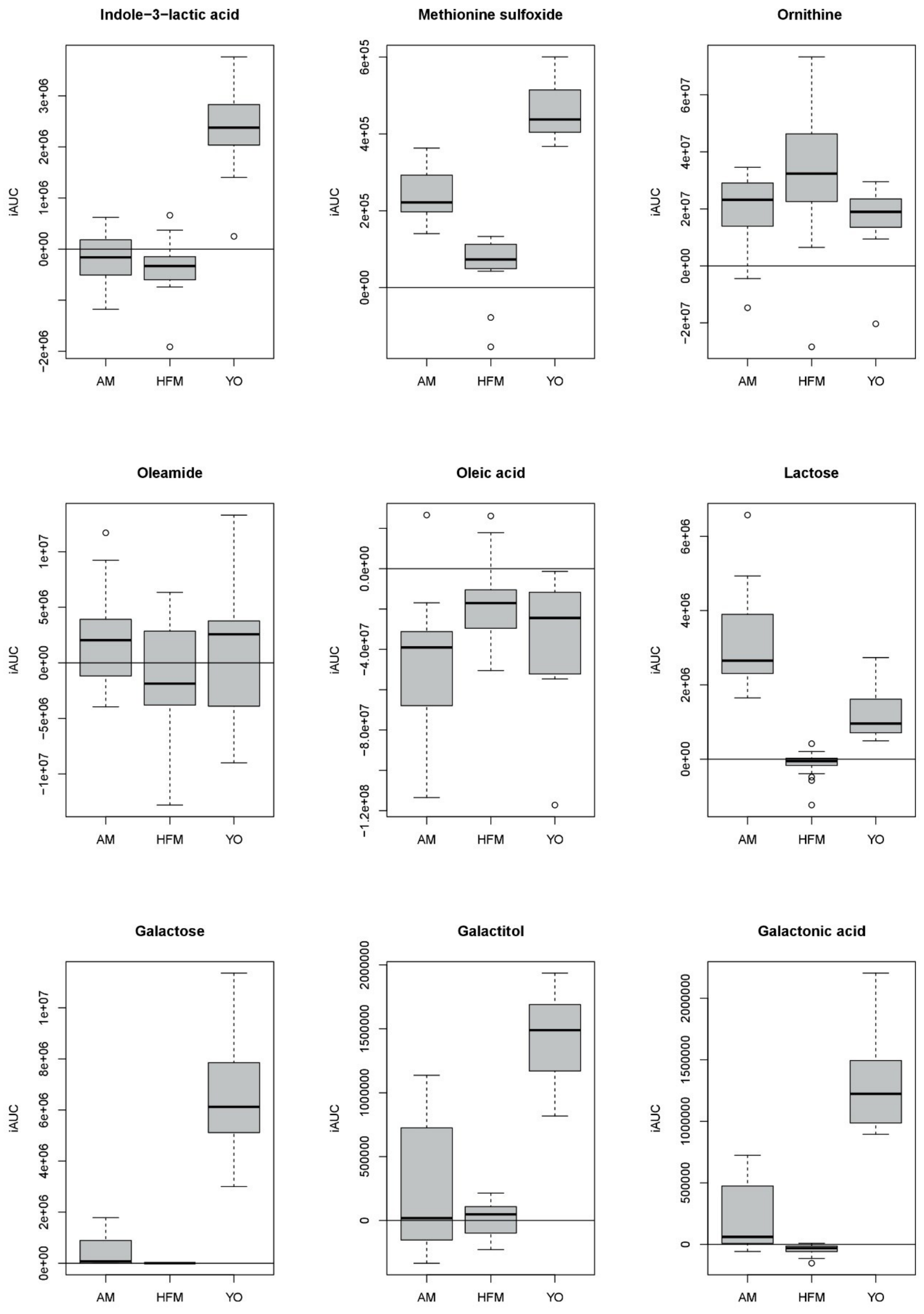
Indole−3−lactic acid
iAUC
−2e+06
0e+00
1e+06
2e+06
3e+06
AM
HFM
YO
Methionine sulfoxide
iAUC
0e+00
2e+05
4e+05
6e+05
AM
HFM
YO
Ornithine
iAUC
−2e+07
0e+00
2e+07
4e+07
6e+07
AM
HFM
YO
Oleamide
iAUC
−1e+07
−5e+06
0e+00
5e+06
1e+07
AM
HFM
YO
Oleic acid
iAUC
−1.2e+08
−8.0e+07
−4.0e+07
0.0e+00
AM
HFM
YO
Lactose
iAUC
0e+00
2e+06
4e+06
6e+06
AM
HFM
YO
Galactose
iAUC
0e+00
2e+06
4e+06
6e+06
8e+06
1e+07
AM
HFM
YO
Galactitol
iAUC
0
500000
1000000
1500000
2000000
AM
HFM
YO
Galactonic acid
iAUC
0
500000
1000000
1500000
2000000
AM
HFM
YO

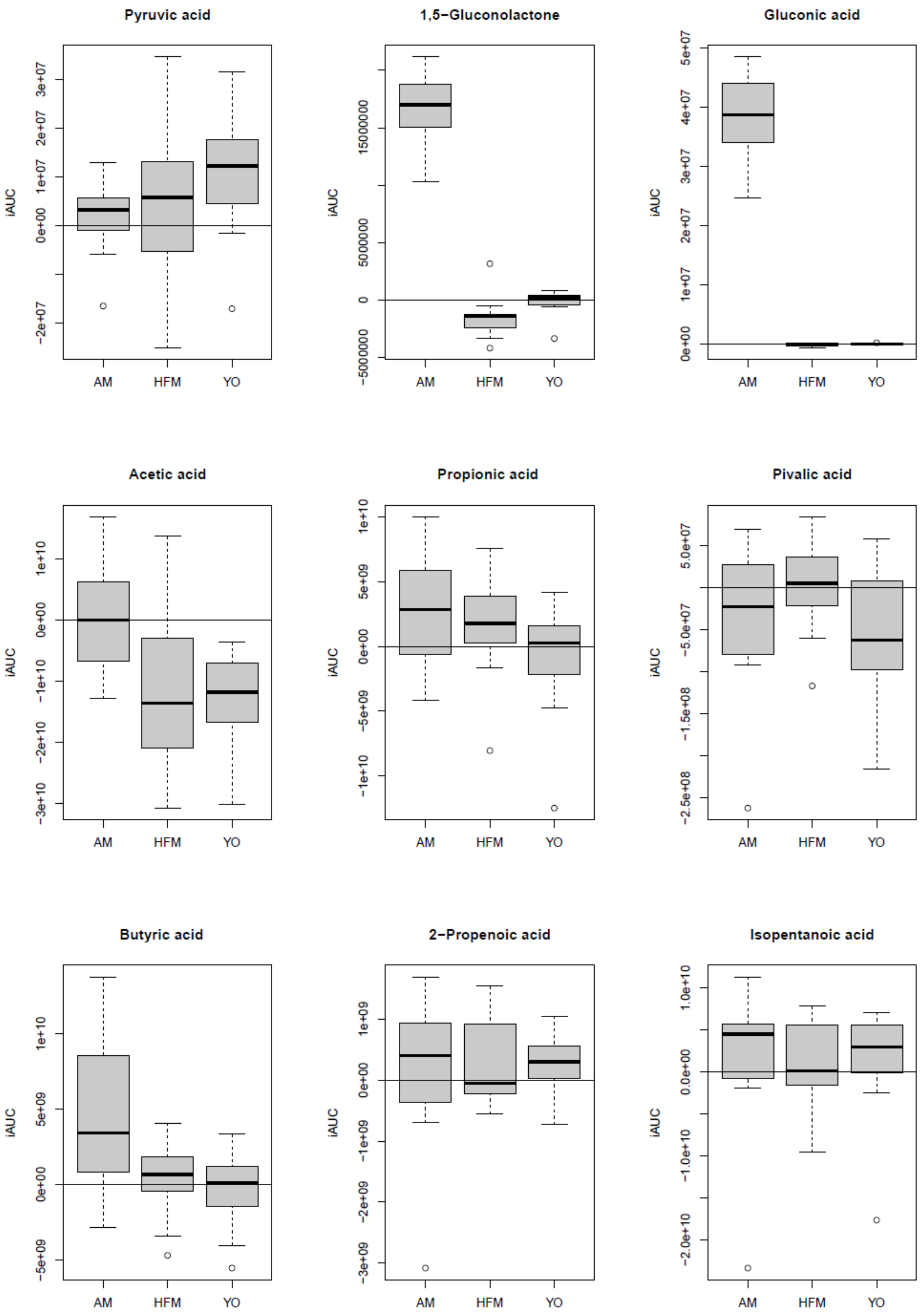
Pyruvic acid
iAUC
3e+07
2e+07
1e+07
0e+00
-2e+07
AM
HFM
YO
1,5-Gluconolactone
iAUC
15000000
5000000
0
-5000000
AM
HFM
YO
Gluconic acid
iAUC
5e+07
4e+07
3e+07
2e+07
1e+07
0e+00
AM
HFM
YO
Acetic acid
iAUC
1e+10
0e+00
-1e+10
-2e+10
-3e+10
AM
HFM
YO
Propionic acid
iAUC
1e+10
5e+09
0e+00
-5e+09
-1e+10
AM
HFM
YO
Pivalic acid
iAUC
5.0e+07
-5.0e+07
-1.5e+08
-2.5e+08
AM
HFM
YO
Butyric acid
iAUC
1e+10
5e+09
0e+00
-5e+09
AM
HFM
YO
2-Propenoic acid
iAUC
1e+09
0e+00
-1e+09
-2e+09
-3e+09
AM
HFM
YO
Isopentanoic acid
iAUC
1.0e+10
0.0e+00
-1.0e+10
-2.0e+10
AM
HFM
YO

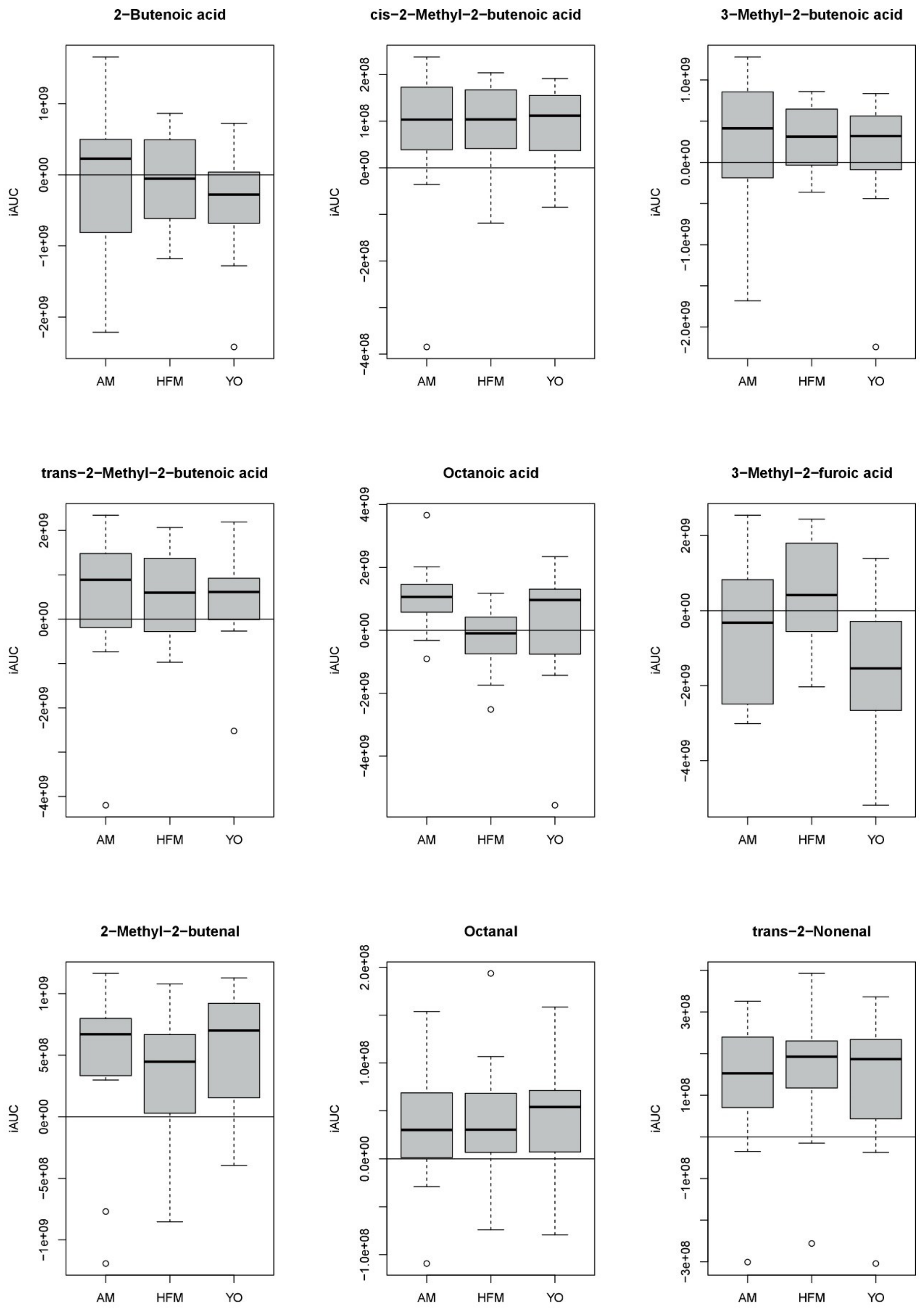
2−Butenoic acid
cis−2−Methyl−2−butenoic acid
3−Methyl−2−butenoic acid
trans−2−Methyl−2−butenoic acid
Octanoic acid
3−Methyl−2−furoic acid
2−Methyl−2−butenal
Octanal
trans−2−Nonenal
iAUC
AM
HFM
YO

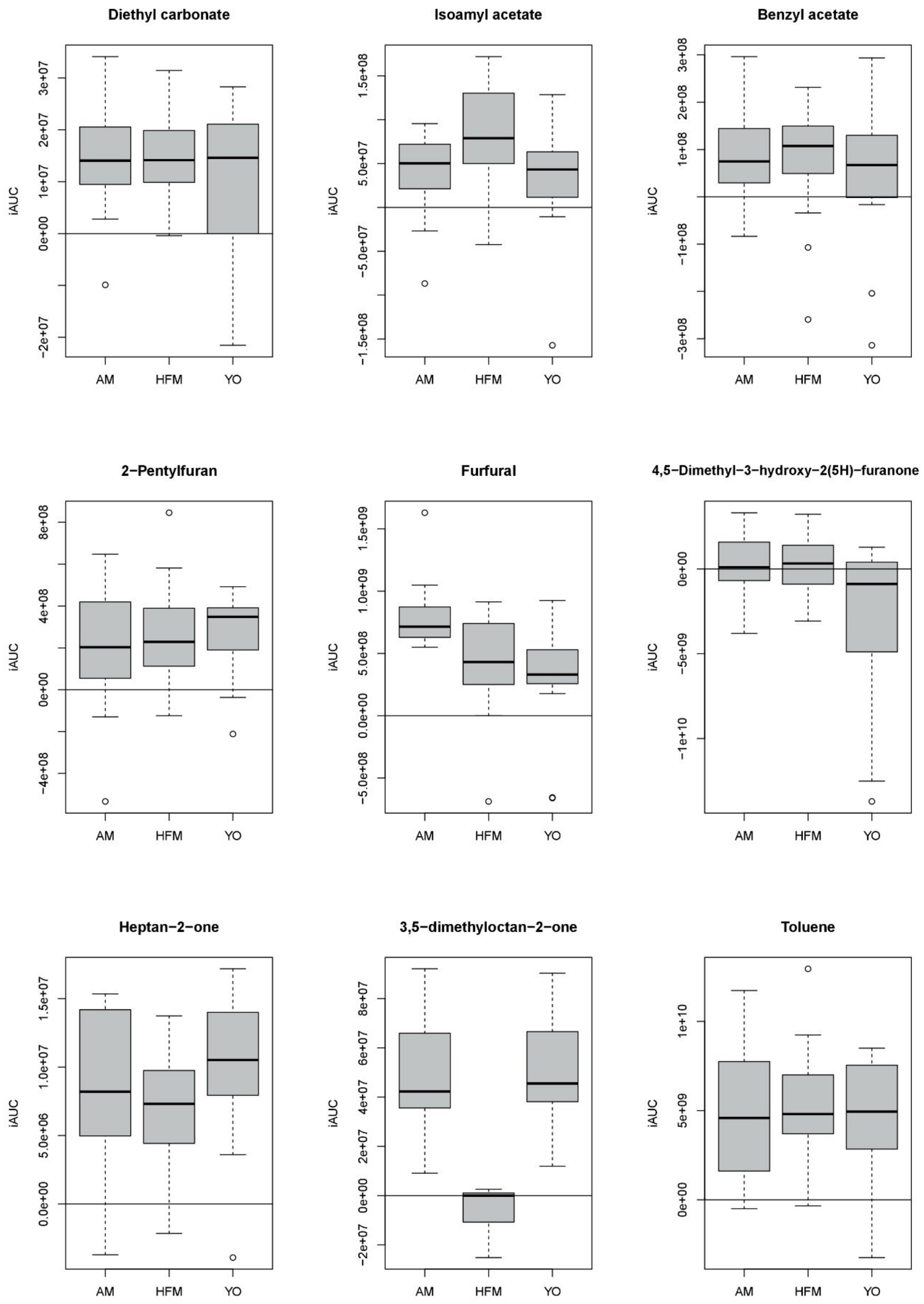
Diethyl carbonate
Isoamyl acetate
Benzyl acetate
2−Pentylfuran
Furfural
4,5−Dimethyl−3−hydroxy−2(5H)−furanone
Heptan−2−one
3,5−dimethyloctan−2−one
Toluene
iAUC
AM
HFM
YO
−2e+07 0e+00 1e+07 2e+07 3e+07
−1.5e+08 −5.0e+07 5.0e+07 1.5e+08
−3e+08 −1e+08 1e+08 2e+08 3e+08
−4e+08 0e+00 4e+08 8e+08
−5.0e+08 0.0e+00 5.0e+08 1.0e+09 1.5e+09
−1e+10 −5e+09 0e+00
0.0e+00 5.0e+06 1.0e+07 1.5e+07
−2e+07 0e+00 2e+07 4e+07 6e+07 8e+07
0e+00 5e+09 1e+10

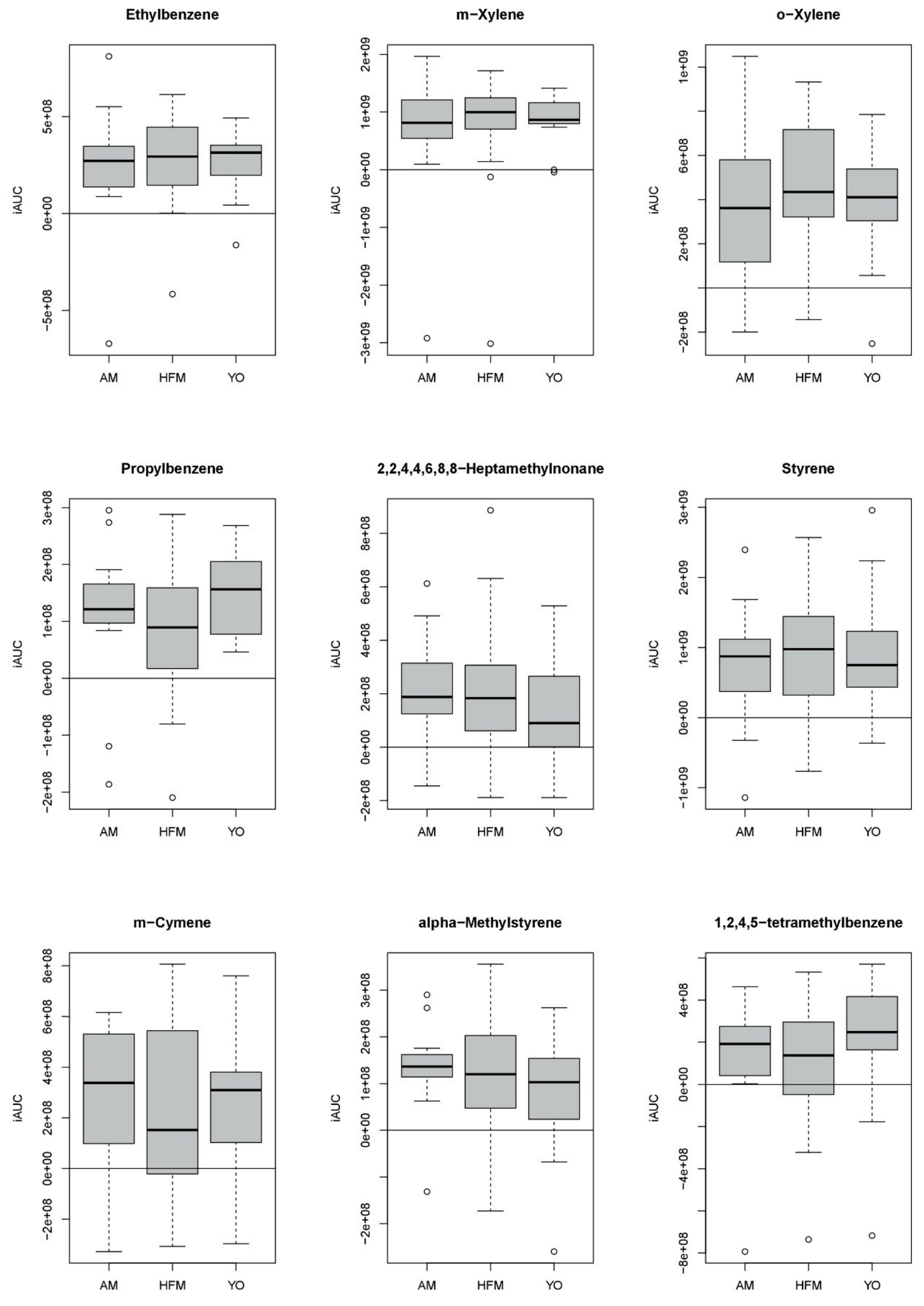
Ethylbenzene
iAUC
-5e+08
0e+00
5e+08
AM
HFM
YO
m−Xylene
iAUC
-3e+09
-2e+09
-1e+09
0e+00
1e+09
2e+09
AM
HFM
YO
o−Xylene
iAUC
-2e+08
2e+08
6e+08
1e+09
AM
HFM
YO
Propylbenzene
iAUC
-2e+08
-1e+08
0e+00
1e+08
2e+08
3e+08
AM
HFM
YO
2,2,4,4,6,8,8−Heptamethylnonane
iAUC
-2e+08
0e+00
2e+08
4e+08
6e+08
8e+08
AM
HFM
YO
Styrene
iAUC
-1e+09
0e+00
1e+09
2e+09
3e+09
AM
HFM
YO
m−Cymene
iAUC
-2e+08
0e+00
2e+08
4e+08
6e+08
8e+08
AM
HFM
YO
alpha−Methylstyrene
iAUC
-2e+08
0e+00
1e+08
2e+08
3e+08
AM
HFM
YO
1,2,4,5−tetramethylbenzene
iAUC
-8e+08
-4e+08
0e+00
4e+08
AM
HFM
YO

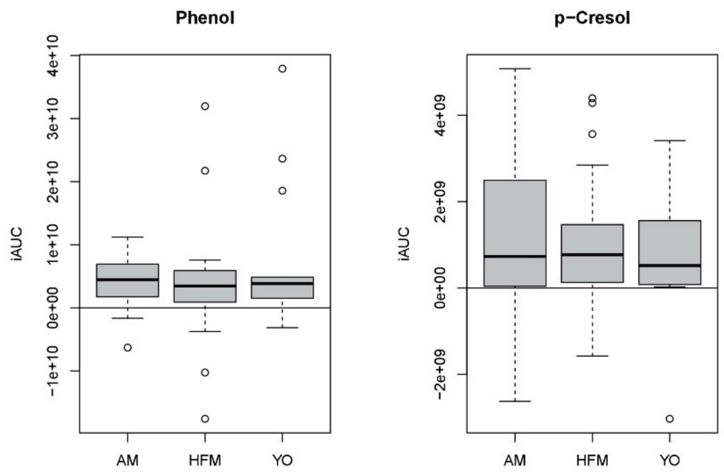

Figure S1: Postprandial response (iAUC) boxplots of 65 postprandial active compounds in serum after consumption of milk (AM), yogurt (YO), and high fat meal (HFM).
